# Comprehensive Screening of Cell Surface Markers Expressed by Adult-Derived Human Liver Stem/Progenitor Cells Harvested at Passage 5: Potential Implications for Engraftment

**DOI:** 10.1155/2016/9302537

**Published:** 2016-11-13

**Authors:** Pierre-Edouard Dollet, Joachim Ravau, Floriane André, Mustapha Najimi, Etienne Sokal, Catherine Lombard

**Affiliations:** Laboratory of Pediatric Hepatology and Cell Therapy, Institut de Recherche Expérimentale et Clinique (IREC), Université Catholique de Louvain, Avenue Mounier 52, 1200 Bruxelles, Belgium

## Abstract

Mesenchymal stromal cells (MSCs) are known to have potential therapeutic benefits for a number of diseases. However, many studies report low engraftment levels, regardless of the target organ. One possible explanation could be that MSCs do not express the necessary receptors for engraftment. Indeed, MSCs appear to use a similar mechanism to leukocytes to engraft into injured organs, relying on various receptors for rolling, firm adhesion, and transmigration. In this study, we conducted an extensive surface molecule screening of adult-derived human liver stem/progenitor cells (ADHLSC) in an attempt to shed some light on this subject. We observed that ADHLSCs lack expression of most of the costimulatory molecules tested. Furthermore, study of the adhesion molecule profile of ADHLSCs revealed that they do not express selectin ligands or LFA-1 which are, respectively, involved in the rolling process and the firm adhesion. In addition, ADHLSCs slightly express VLA-4 and lose expression of CXCR4 altogether on their surface during culture expansion. However, ADHLSCs express all the integrin couples and matrix metalloproteinases needed to bind and integrate the extracellular matrix once the endothelial barrier is crossed. Collectively, these results suggest that binding to the endothelium may be the critical weak point in the engraftment process.

## 1. Introduction

Mesenchymal stromal cells (MSCs) have been isolated and characterized from various sources (liver, heart, lung, and bone marrow) [1]. Some of them are currently being investigated for cell therapy in the treatment of a wide range of diseases (cancer, heart stroke, inflammatory diseases, and genetic disorders). Our group has previously isolated and characterized stem/progenitor cells from healthy adult human liver (ADHLSCs) [2, 3]. These expandable cells show a hepatomesenchymal phenotype and have the potential to differentiate into hepatocyte-like cells both in vitro and in vivo [2, 4, 5]. ADHLSCs are now in phase 2/3 of clinical trials to treat inborn errors of metabolism of the liver such as urea cycle disorders or Crigler Najjar syndrome.

However, as is the case with most mesenchymal stem/progenitor cell-based therapies, the rate of engraftment of ADHLSCs into the recipient liver remains low [4]. One hypothesis is that donor cells could be cleared by the immune system of the recipient. Our previous studies indicated that ADHLSCs are poorly immunogenic [6, 7], but their immune profile has not yet been completely characterized. In addition, there could be some impairment in the engraftment process itself. A number of studies suggest that the engraftment process of MSCs is similar to that of leukocytes or hematopoietic stem cells (HSCs). The cells pass through a rolling phase, followed by a firm adhesion step, and finally transmigration through the endothelium [8, 9], which takes 10 to 20 minutes for leukocytes and 60 to 120 minutes for MSCs [10]. Unlike leukocytes, MSCs do not express the same number of adhesion molecules to accomplish this engraftment process. First, MSCs do not express the selectin ligands required to slow them down on activated endothelium [11]. Second, they do not express lymphocyte function-associated antigen 1 (LFA-1), which would allow them to bind to intercellular adhesion molecule 1 (ICAM-1) on endothelial cells. However, during inflammation, the activated endothelium secretes stromal cell-derived factor 1 (SDF-1), which increases recruitment of MSCs through C-X-C chemokine receptor type 4 (CXCR4) and can also activate cells and help in the firm adhesion step mediated by very late antigen-4/vascular cell adhesion protein 1 (VLA-4/VCAM) [12, 13]. Transmigration through the endothelium appears to rely on VLA-4/VCAM binding, followed by use of matrix metalloproteinases to integrate into the organ. We have previously shown that ADHLSCs express some adhesion molecules [2, 7], but information was still lacking on a number of key receptors involved in the engraftment process. In addition, the requirements of large scale cultures for clinical use have prompted us to move from culture on collagen-coated flasks and an emergence in the presence of EGF, to culture on CellBIND® plastic, treated to facilitate adhesion.

In the current study, we performed extensive screening of all ADHLSC surface antigens using the BD Lyoplate™ human cell surface marker screening panel following culture in large scale conditions. This screening also allowed us to complete their surface marker characterization, confirm their low expression of immunogenic markers, and shed light on potential weak points in the ADHLSC engraftment process.

## 2. Materials and Methods

### 2.1. ADHLSC Isolation and Culture

The protocol and experiments were approved by the ethics committees of the St Luc's University Hospital and the Faculty of Medicine of the Université Catholique de Louvain. Approval from the Belgian Ministry of Health was obtained for the hepatocytes and hepatic stem cells bank. Written and signed informed consent was also obtained for each human liver used in the current study.

Eight donors were used in the current study (Table 1). ADHLSCs were recovered subsequent to primary culture of the liver parenchymal fraction achieved after two-step collagenase perfusion, filtration, and low-speed centrifugation, as described elsewhere [2]. ADHLSCs were cultured on CellBIND flasks (Corning®) in Dulbecco's modified Eagle's medium (DMEM) containing 4.5 g/L glucose (Invitrogen), supplemented with 10% fetal calf serum (Gibco) and 1% penicillin/streptomycin (Invitrogen), at 37°C in a fully humidified atmosphere (5% CO_2_). Upon reaching 80% confluence, cells were lifted with 0.05% trypsin-EDTA (Invitrogen) and replated at a density of 5000 cells/cm^2^. The viability of the recovered cells was evaluated using the trypan blue dye exclusion method.

### 2.2. Cell Surface Marker Screening by Flow Cytometry Using BD Lyoplate Technology

The BD Lyoplate human cell surface marker screening panel (BD Biosciences, Heidelberg, Germany) was used to characterize cultured ADHLSCs. The kit contains 242 purified monoclonal antibodies to cell surface markers, as well as isotype controls to assess nonspecific backgrounds. Before use, plates containing lyophilized antibodies were centrifuged at 300 ×g for 5 minutes. The antibodies were then reconstituted in 110 *μ*L of sterile Dulbecco's Phosphate-Buffered Saline (DPBS).

The assay was performed on five donors, according to the manufacturer's instructions. Briefly, ADHLSCs were harvested at passage 5 using 0.05% trypsin-EDTA. After washing in DPBS, cells were resuspended in Pharmingen stain buffer containing 5 mM EDTA at a concentration of 1.25 × 10^6^ cells/mL. Eighty microliters of cell suspension per well was then transferred to 96-well plates and stained with 20 *μ*L of specific primary antibodies for 30 minutes on ice. Thereafter, the cells were washed twice with Pharmingen stain buffer + 5 mM ETDA and stained with 100 *μ*L of Alexa Fluor 647-labeled anti-mouse or anti-rat secondary antibody (diluted 1 : 200 in Pharmingen stain buffer + 5 mM EDTA) for 30 minutes on ice. After washing, the cells were fixed with BD cytofix fixation buffer and transferred from the 96-well plates to single BD FACS tubes. Fluorescence was measured with a BD FACSCanto II cytometer on 10,000 cells using FACSDiva software.

For analysis, background fluorescence was set manually for each sample based on its appropriate isotype using FlowJo software. Results are expressed as a percentage of positive cells in the population or median fluorescence intensity (MFI).

### 2.3. Analysis of CD184 and CD90 Expression by Flow Cytometry

For cell surface staining, liver cells were first incubated with DPBS-bovine serum albumin (BSA) 1.5% for 20 minutes at 4°C to prevent nonspecific binding. Next, the cells were washed with DPBS-BSA 1.5% and stained with 5 *μ*L of PE rat anti-human CD184, APC mouse anti-human CD90, or their respective isotypes (BD Biosciences) for 30 minutes on ice. Finally, the cells were washed and fixed using a stabilizing fixative (BD Biosciences). For intracellular staining, liver cells were fixed and permeabilized with 200 *μ*L of cytofix/cytoperm buffer (BD Biosciences) for 20 minutes at 4°C. The cells were then washed with perm/wash buffer and stained with PE rat anti-human CD184 or its isotype diluted in perm/wash for 30 minutes on ice. Next, the cells were washed twice and fixed with stabilizing fixative (BD Biosciences). Fluorescence was measured with a BD FACSCanto II cytometer on 10,000 cells using the FACSDiva software. Data analyses were performed with FlowJo software.

### 2.4. Immunofluorescence

ADHLSCs were plated at passage 4 on 8-chamber slides (BD Biosciences) at a density of 5,000 cell/cm^2^. Upon reaching 70% confluence, they were fixed with 4% paraformaldehyde for 15 minutes. After 2 washes with DPBS, ADHLSCs were blocked with DPBS-BSA 5% for 1 hour. Some of the samples were permeabilized with Triton 0.1% DPBS-BSA 1.5% buffer (BD Biosciences) for 20 minutes at 4°C. The cells were then stained with a PE rat anti-human CD184 for 2 hours at 4°C (BD Biosciences) and rinsed 3 times with DPBS. Finally, samples were embedded in ProLong Gold with DAPI (BD biosciences). Pictures were taken with an Axio Imager + ApoTome (Zeiss) at a 20x objective and analyzed with AxioVision software.

### 2.5. Real-Time PCR

Total RNA was extracted from 4 ADHLSC donors at passage 5 using TriPure isolation reagent (Roche, Mannheim, Germany), following the manufacturer's instructions. Briefly, 1.5 × 10^6^ cells were homogenized in TriPure reagent, mixed with chloroform, shaken vigorously for 15 second, and centrifuged at 12,000 ×g for 15 minutes at 4°C. RNA in the upper aqueous phase was precipitated by isopropanol, washed in 75% ethanol, air-dried, and dissolved in RNase-free water. RNA samples were stored at −80°C after quantification with a NanoDrop 2000 spectrophotometer (Thermo Scientific).

cDNA was synthesized from 1 *μ*g of total RNA by reverse transcription polymerase chain reaction (RT-PCR) using a high-capacity kit (Applied Biosystems). Thereafter, 10 ng of RT product was deposited in each well of a TaqMan® array human extracellular matrix and adhesion molecules (Invitrogen), as instructed by the manufacturer. Plates were read using the Applied Biosystems StepOnePlus real-time PCR system. The PCR data were normalized with the housekeeping gene GUSB (Glucuronidase Beta).

### 2.6. Statistical Analysis

Experimental data were expressed as the mean ± SEM and were analyzed using two-way analysis of variance (ANOVA). Values of *P* < 0.05 were considered to be of statistical significance.

## 3. Results and Discussion

### 3.1. Mesenchymal Phenotype of ADHLSCs

**Figure 1:**
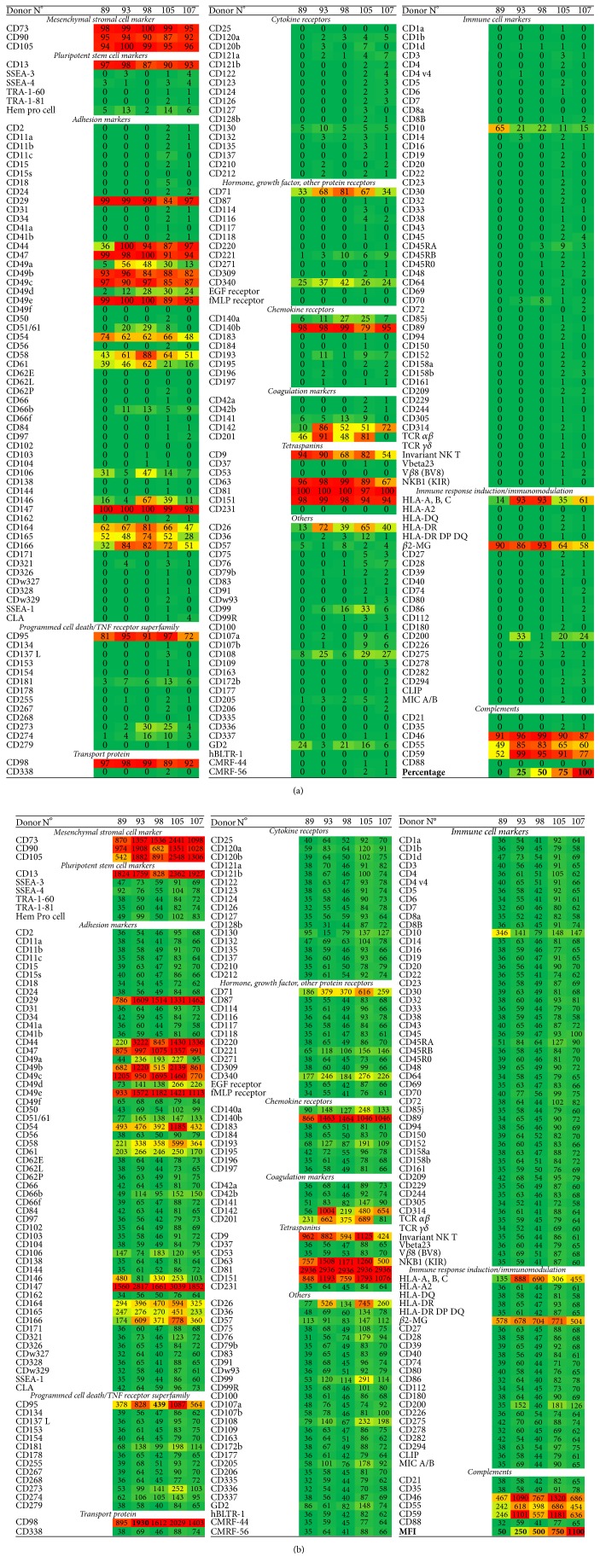
Heat map of cell surface marker expression of ADHLSCs using the BD Lyoplate. Results are expressed as percentage of positive cells (a) and in median fluorescence intensity of the total population (MFI) (b). For reference, isotype controls showed an average MFI of 60 (*n* = 5).

Screening performed on five donors using the BD Lyoplate confirmed that ADHLSCs harvested at P5 express CD73, CD90, and CD105 but lack CD11b, CD14, CD19, CD79*β*, CD45, and HLA-DR expression (Figure 1 and Table 2), which are characteristics required by the International Society for Cellular Therapy to be recognized as MSCs [14]. CD90 expression was slightly lower than the 95% required, probably due to the moderate stain index of the fluorochrome provided (AF-647) resulting in a suboptimal resolution of the peaks. These results correlate with data previously shown by our team, aside from the expression of CD105, which seems to be increased after culture on CellBIND [2]. Although it would have been interesting to evaluate how the expression of the different markers tested here evolves throughout the culture process, we have focused on P5 as it is the passage currently used in the clinic. In addition, we have used a broad range of donors to represent the diversity of donors used in the clinic. Despite the differences in age and cause of death, it has to be noted that the general characteristics regarding the presence or absence of a certain marker are fairly consistent among the group; the variability lies in the degree of expression. More studies would be needed to determine the influence of donor age and cause of death on the level of expression of the markers tested here. Interestingly, a screening performed by Baer et al. [15] on adipose-derived stromal/stem cells (ASCs) using the same assay revealed a phenotype close to what we found for ADHLSCs, with the exception of a few markers expressed by ASCs and absent from ADHLSCs and bone marrow derived MSCs (CD34, CD36). ASCs also express CD91 which is absent from ADHLSCs (Table 2).

ADHLSCs did not appear to express markers of pluripotent stem cells, except for CD13, which was expressed at very high levels, confirming our previous results (Figure 1) [2]. However, it should be noted that, despite CD13 being initially described in relation to pluripotent stem cells, subsequent studies suggested an additional role for the molecule, including cell adhesion [16, 17]. Interestingly, it has been implicated in the adhesion of monocytes to the endothelium.

### 3.2. Immunogenic Phenotype

ADHLSCs did not express any immune cell markers, as expected. However, these cells did express human leukocytes antigens (HLA) A, B, and C and *β*2-microglobulin, which are components of MHC class I, as do all nucleated cells, and could therefore be the target of cytotoxic T cells (Figure 1). Moreover, ADHLSCs did not express any of the other proteins tested that could trigger an immune response during infusion (Figure 1). These results seem to be in accordance with our previous reports that ADHLSC are poorly immunogenic [6]. In fact, our data suggest that these cells are immunosuppressive. However, the list of immunomodulatory markers tested here is not exhaustive and the immunosuppression assays performed were limited to the inhibition of PHA/IL-2 stimulated T cells. Therefore, further research would have to be performed to confirm the poorly immunogenic phenotype of ADHLSCs and better understand their effect on immune cells. In addition, our results suggest that ADHLSCs could be protected against the complement cascade following infusion thanks to the expression of CD46 and CD55, which may inactivate proteins C3b and C4b, and the expression of CD59, which can block complement protein C9. Nevertheless, expression of CD95 (Fas receptor) by ADHLSCs renders cells susceptible to apoptosis through ligation by a secreted Fas ligand protein or contact with a Fas ligand-bearing adjacent cell [18].

### 3.3. Procoagulant Phenotype

ADHLSCs have been shown to have procoagulant activity due to the presence of tissue factor (CD142) [19], a member of the coagulation cascade required for thrombin formation, expression of which we confirm in this study. We also show that ADHLSCs do not express CD42 (a or b), which are platelet surface glycoproteins, or CD141 (thrombomodulin). They do, however, express CD201, also known as activated protein C receptor, which plays a role in anticoagulation (Table 2). It is noteworthy that the level of expression of CD141 and CD142 varied from donor to donor, suggesting that the pro- or anticoagulant properties of ADHLSCs are likely to be donor-dependent.

### 3.4. Tetraspanin, Cytokine, Chemokine, Hormone, and Growth Factor Receptor Expression

Our study shows that ADHLSCs express several tetraspanin family members, such as CD9, CD63, CD81, and CD151. Although their function is not entirely known, they appear to play a role in signal transduction. A transporter of amino acids (CD98) was also detected, as was the DPPIV enzyme, also known as CD26, which is highly expressed in the liver [20].

Only a few cytokine, chemokine, hormone, and growth factor receptors were found. CD71 (transferrin receptor protein 1) and CD140b (beta-type platelet-derived growth factor receptor) were detected on the surface of ADHLSCs at passage 5 (Table 2). However, it is possible that some receptors became internalized during the culture process.

### 3.5. Adhesion Proteins

**Figure 2:**
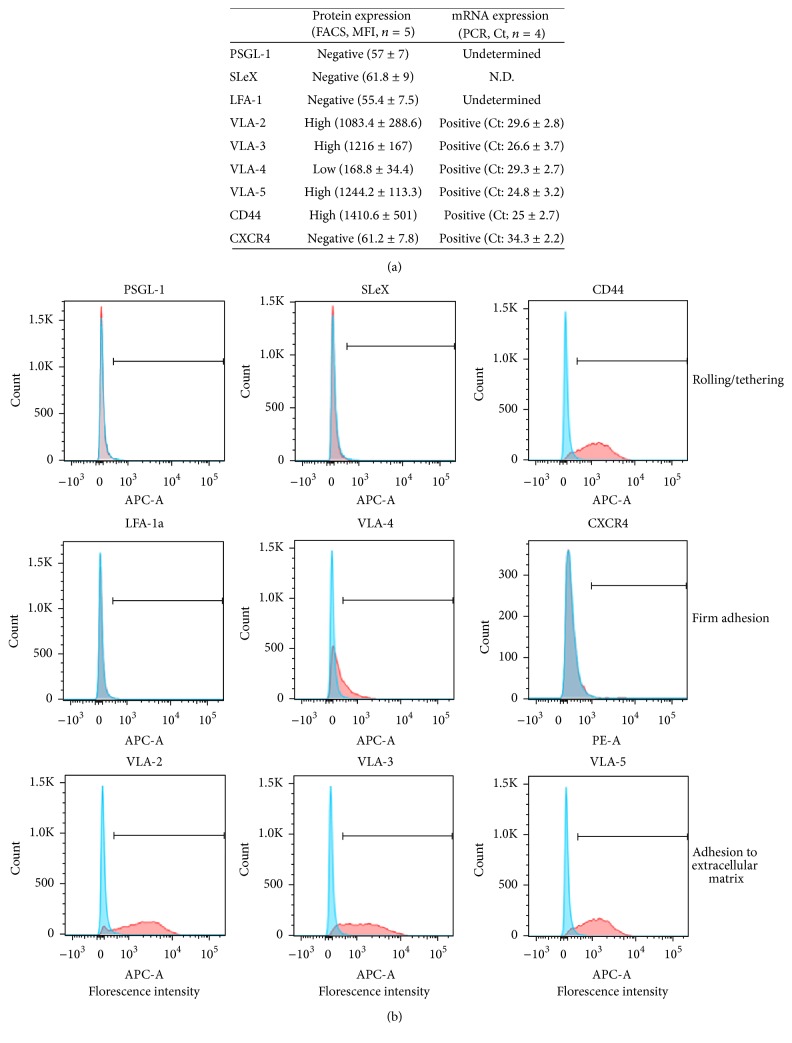
Antigen and mRNA expression of the main molecules involved in the engraftment process. (a) Results are expressed in mean (± standard error of the mean) of median fluorescence intensity for the protein expression (FACS; *n* = 5 donors) and of Ct values for the mRNA expression (real-time PCR; *n* = 4 donors). (b) Histograms of the antigens PSGL-1, LFA-1*α*, VLA-4, and VLA-5; analyses with FlowJo. Red histograms represent cells stained for the antigen of interest versus cells stained with appropriate isotype controls in blue.

This study was designed in part to evaluate the expression of adhesion proteins that would allow ADHLSCs to bind to the endothelium and extracellular matrix during the engraftment process. In order to reach the parenchyma of the organ following peripheral injection, MSCs must behave like leukocytes during inflammation: first, they must decrease their speed on the endothelium with the help of selectin ligands; second, they must adhere firmly to endothelial proteins such as ICAM and VCAM-1 using integrin dimers like VLA-1 (*α*L*β*2) and VLA-4 (*α*4*β*1), respectively. BD Lyoplate screening was followed by evaluation of the expression of some of the proteins of interest at the mRNA level using the TaqMan array for human extracellular matrix and adhesion molecules. This was done in order to distinguish molecules that are completely absent from those that are absent or barely expressed at the protein level, but expressed at the mRNA level. As shown in Figures 1 and 2, ADHLSCs, like most MSCs, did not express either CD162 (PSGL-1) or sialyl-Lewis X (SLeX), a tetrasaccharide component of PSGL-1 required to bind E-selectin, on their surface. Real-time PCR analysis proved that they were not expressed at the mRNA level either. Sarkar et al. were able to make MSCs roll on P-selectin and activated endothelial cells by linking a sialyl-Lewis X group to the cell surface via a biotin streptavidin bridge, thereby decreasing the rolling velocity of cells in vivo [11]. Interestingly, CD44, which is considered by some [8, 9] as an alternative for the rolling/adhesion process, is highly expressed by ADHLSCs. However, in these publications, CD44 had to be engineered with the fucosyltransferase enzyme to have the ability not only to bind selectins, but also to improve cell engraftment of BM-MSCs in NOD/SCID mouse bone marrow after 24 hours [21, 22]. Because fucosyltransferase IV (SSEA-1) is not expressed by ADHLSCs (Figure 1) [21], CD44 may not be functional as an adhesion protein under noninflammatory conditions [21]. However, cell adhesion may depend on the organ targetted and its inflammatory status. Indeed, reports have shown that while neutrophils rely on tethering and rolling followed by firm adhesion to integrate most tissues, in the inflamed liver, their adhesion to the sinusoidal endothelium relies on direct binding of their CD44 to the hyaluronan expressed by the endothelial cells [23, 24]. Therefore, the lack of PSGL1 and other adhesion molecules involved in rolling and firm adhesion may be overcome by the high expression of CD44 at the surface of ADHLSCs. In addition, binding to hyaluronan in the sinusoids may help keep the cells in the liver and limit their dissemination to nontargetted organs. Our results also reveal that ADHLSCs, like all MSCs, do not express VLA-1 at the protein or mRNA level, but they do show a slight expression of VLA-4 on their surface (average MFI of 168.8 for 5 donors) (Figures 1 and 2) [12, 25], despite a constitutive expression at the mRNA level (Figure 2). In addition, ADHLSCs show high expression of all the integrins needed to bind to the extracellular matrix once they have passed through the endothelium: VLA-2 to bind to collagen, VLA-3 to bind to laminin, and VLA-5 to bind to fibronectin [26]. However, even if ADHLSCs express most of the integrins needed to bind to the extracellular matrix, low expression of VLA-4, which appears to be the most important protein for the rolling/adhesion process and binding to the endothelium, and the absence of selectin ligand may be sufficient to explain the low engraftment rates, as cells are unable to stop and attach to the endothelium [12, 27]. On the other hand, upregulation of integrin alpha 4 by an adenovirus vector was shown to increase cell engraftment by 25% in the bone marrow of C57BL mice [28]. Further research is currently under way to determine the importance of these molecules in the adhesion of ADHLSCs to the endothelium and the extracellular matrix.

### 3.6. CXCR4 Expression

**Figure 3:**
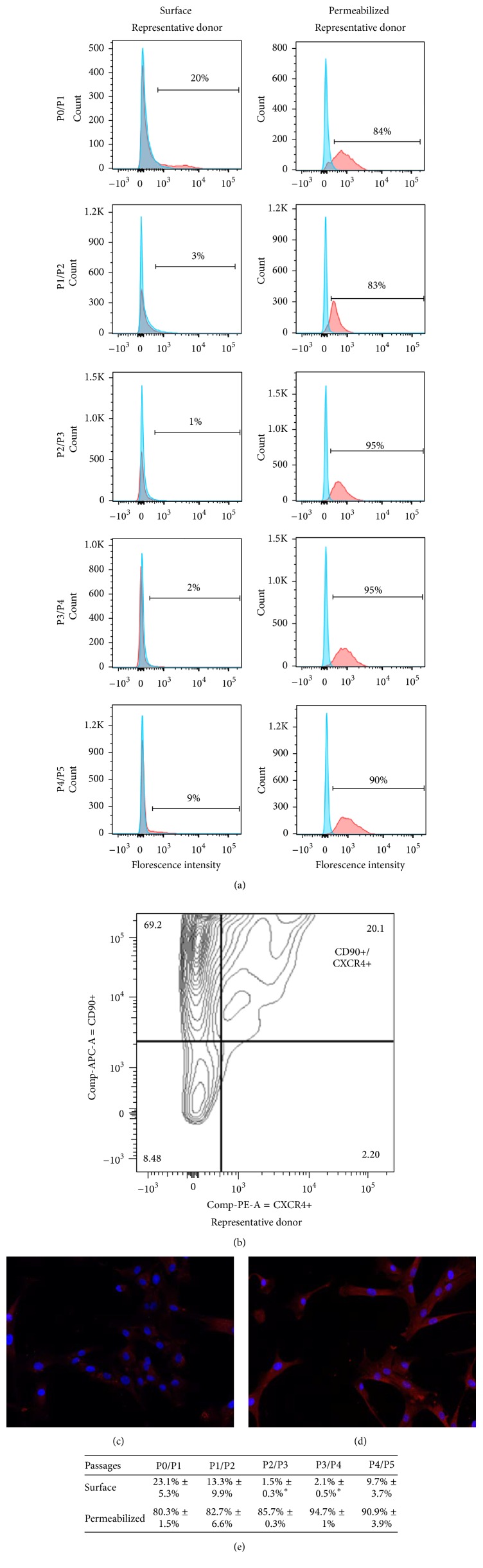
CXCR4 expression of ADHLSCs. (a) CXCR4 surface and internalized expression from passages 1 to 4 by flow cytometry. (b) Contour plot of double staining for CD90 and CXCR4 at passage 1. Representative immunofluorescence pictures of CXCR4 staining at passage 4 for permeabilized (d) and nonpermeabilized (c) ADHLSCs. (e) Cell surface and intracellular CXCR4 expression (± standard error of the mean) from passages 1 to 5 (*n* = 4 donors); significant differences have been found on the surface expression between P0/P1 and P2/P3 and P3/P4 (^*∗*^ *P* < 0.05).

Another important protein involved in the homing process is the 7-transmembrane G-coupled receptor CXCR4. CXCR4 has been described as the major receptor involved in the engraftment/homing process of HSCs and MSCs. At injury sites, CXCR4 binds released SDF-1, which facilitates cell migration to organs [13]. Use of the CXCR4 antagonist AMD3100 during cell infusion was shown to inhibit migration of MSCs to the acutely injured kidneys [29, 30]. However, several groups have reported that CXCR4 expression decreases rapidly after MSCs isolation and only a very small percentage of cells or none at all express CXCR4 after a few passages [31, 32]. In fact, in vitro expansion of MSCs induces progressive internalization of CXCR4 as a way for cells to adapt to culture conditions, to the point where there is no CXCR4 remaining on their surface [12, 33–35]. Considering that ADHLSCs emerge from the parenchymal fraction of the adult liver after about one month in culture and then need several more weeks to reach passages 4 to 6 at which time they are traditionally used for experiments, we suspected that the same phenomenon may be taking place. We therefore evaluated surface expression of CXCR4 at each passage by flow cytometry and found that all donors tested showed a surface receptor expression around 23.1%  ± 5.3% at passage 1 (Figure 3(e)), which decreased significantly until 2.1%  ± 0.5% at the fourth passage. However, when the cells were permeabilized, 80.3%  ± 1.5% of the cell population expressed CXCR4 at passage 1, and the expression remained at 94.7%  ± 1% at passage 4, suggesting that a large portion of the population had already started to internalize CXCR4 (*n* = 4). To verify that the CXCR4-positive cells were indeed ADHLSCs, double staining of CXCR4 with CD90 was performed on three donors (Figure 3(b)). These results show that ADHLSCs have a pattern of CXCR4 expression identical to that of BM-MSCs, which could be a specific characteristic of MSCs. Rombouts and Ploemacher found that the engraftment capacity of freshly isolated MSCs was higher than that of cultured MSCs even after only 24 hours of culture [36]. Consequently, some research groups have decided to induce externalization of CXCR4 on the surface of MSCs, a key point to enhance MSC homing. Different methods have been used to upregulate CXCR4, such as culture with valproic acid (VPA) [37, 38], C1q [39], SDF-1 [40], or a cytokine cocktail [34], culture under hypoxic conditions [41], or even direct transduction with a gene encoding the receptor [42]. In our experience, neither the cocktail of cytokines described by Shi et al., nor preincubation of ADHLSCs with SDF-1 [40] had any effect on CXCR4 externalization. However, in a patient with factor VIII deficiency infused intravenously with ADHLSCs at passages 4 and 5, we found cells engrafted at the injury site (hemarthrosis), highlighting the capacity of ADHLSCs to engraft into inflammatory areas (unpublished data).

## 4. Conclusion

In conclusion, even if we cannot completely rule out that the low levels of engraftment observed following infusion are due to cell clearance, our data suggest that ADHLSCs are poorly immunogenic overall. However, adhesion molecules expressed by the cells appear to point to an impaired capacity to bind to the endothelium, which could potentially lead to lower engraftment. In addition, our findings indicate that the pattern of expression of some of these proteins may result from the culture process. Further studies are needed to evaluate the precise impact of culture conditions on the expression of cell surface markers by ADHLSCs and determine whether modifying the culture process could improve cell engraftment.

## Figures and Tables

**Table 1:** Characteristics of the 8 liver donors from which ADHLSCs were isolated.

Donor number	Age	Gender	Reason of death	Blood group
15	25 years	M	/	A+
89	3 days	M	Respiratory	A+
93	2 years	F	Metabolic disease	O+
98	7 days	M	Cardiorespiratory arrest	O−
105	46 years	F	Traumatism	B+
107	4 days	F	Nonketotic hyperglycemia	O+
115	3 months	M	Meningitis	O+
116	6 days	F	Neonatal asphyxia	O+

**Table 2:** Expression of MSC markers of ADHLSCs and comparison with BM-MSC and ASC.

Markers	ADHLSC	BM-MSC [14, 15]	ASC [15]
CD73	98.3% (95.0–99.7)	≥95% (guideline)	≥95%
CD90	91.5% (86.9–95.0)	≥95% (guideline)	≥95%
CD105	96.7% (93.8–99.6)	≥95% (guideline)	≥95%
CD11b	0.7% (0.0–2.0)	≤2% (guideline)	≤2%
CD14	2.0% (0.0–3.26)	≤2% (guideline)	≤2%
CD19	0.5% (0.0–2.0)	≤2% (guideline)	≤2%
CD45	1.1% (0.0–2.8)	≤2% (guideline)	≤2%
HLA-DR	0.8% ± 0.5	≤2% (guideline)	≤2%

CD34	1.3% (0.1–2.6)	0.1% (0.0−0.1)	9.0% (5.1–30.1)
CD36	3.5% (0.0–12.0)	0.1% (0.0−0.2)	11.5% (4.8–13.4)
CD91	0.7% (0.0−1.7)	N.D.	47.6% (12.7–87.4)
CD140b	93.8% (99.4–78.6)	54.3% (45.9–87.5)	79.9% (49.2–87.5)
CD141	7.2% (4.2–12.8)	N.D.	≥95%
CD201	53.3% (0.17–81.1)	2.0% (1.8–4.8)	13.6% (9.5–25.6)
